# Supplementary material for: The association of diet quality with the mental health of students during their transition to university
Source: PLoS One. 2024 Oct 15;19(10):e0312123. doi: 10.1371/journal.pone.0312123 (PMC11478860; doi:10.1371/journal.pone.0312123)
Supplement: Attachment — Submitted filename: PONE-D-24-28220.pdf [file pone.0312123.s001.pdf]

PLOS ONE

The association of diet quality with the mental health of students during their transition to university

--Manuscript Draft--

**Manuscript Number:**	PONE-D-24-28220
**Article Type:**	Research Article
**Full Title:**	The association of diet quality with the mental health of students during their transition to university
**Short Title:**	Diet and mental health of university students
**Corresponding Author:**	Solomis Solomou<br>Lancaster University<br>Lancaster, UNITED KINGDOM OF GREAT BRITAIN AND NORTHERN IRELAND
**Keywords:**	Mental Health; Diet Quality; Students; University; Transition
**Abstract:**	University students are at risk of experiencing mental health and diet quality problems during their transition to university. This study aimed to examine the bidirectional associations between the diet quality and mental health of students during their transition to universities in the UK, and the impact of the transition on diet quality and mental health. The study adopted a cross-sectional design and took place during the first semester of year 2021-2022. Participants were first year undergraduate students at four UK universities, living at student halls. They were invited to participate by completing online surveys. Results showed that a healthy diet was associated with good mental health (b = -3.46, 95% CI [-6.14, -.78[illegible]nfavourable mental health was associated with having an unhealthy diet (b = -.01[illegible]% CI [-.02, -.00]). Transition did not moderate the relationship between diet quality and mental health of students, or vice versa (ps> .05). Preliminary analyses showed a significant direct effect of good quality of transition to university on good mental health (b = -1.51, 95% CI [-1.88, -1.13]), but did not have an indirect effect via diet quality; there were no significant direct or indirect effects on diet quality, however larger studies are needed to replicate these preliminary analyses. Our current findings can inform university policies and health education research, and further research is needed to determine whether interventions to improve diet quality at the university level could reduce mental health issues, and whether interventions to support students under stress may lead to healthier dietary habits.
**Order of Authors:**	Solomis Solomou
	Heather Robinson
	Guillermo Perez Algorta
**Additional Information:**	
**Question**	**Response**
**Financial Disclosure**<br><br>Enter a financial disclosure statement that describes the sources of funding for the work included in this submission. Review the submission guidelines for detailed requirements. View published research articles from *PLOS ONE* for specific examples.<br><br>This statement is required for submission and **will appear in the published article** if	The author(s) received no specific funding for this work.

the submission is accepted. Please make sure it is accurate.<br><br>**Funded studies**<br>Enter a statement with the following details:<br>• Initials of the authors who received each award<br>• Grant numbers awarded to each author<br>• The full name of each funder<br>• URL of each funder website<br>• Did the sponsors or funders play any role in the study design, data collection and analysis, decision to publish, or preparation of the manuscript?<br><br>Did you receive funding for this work?	
**Competing Interests**<br><br>Use the instructions below to enter a competing interest statement for this submission. On behalf of all authors, disclose any competing interests that could be perceived to bias this work—acknowledging all financial support and any other relevant financial or non-financial competing interests.<br><br>This statement is required for submission and **will appear in the published article** if the submission is accepted. Please make sure it is accurate and that any funding sources listed in your Funding Information later in the submission form are also declared in your Financial Disclosure statement.<br><br>View published research articles from *PLOS ONE* for specific examples.	The authors have declared that no competing interests exist.

Powered by Editorial Manager® and ProduXion Manager® from Aries Systems Corporation

**NO authors have competing interests**<br><br>Enter: *The authors have declared that no competing interests exist.*<br><br>**Authors with competing interests**<br><br>Enter competing interest details beginning with this statement:<br><br>*I have read the journal's policy and the authors of this manuscript have the following competing interests: [insert competing interests here]*<br><br>* typeset	
**Ethics Statement**<br><br>Enter an ethics statement for this submission. This statement is required if the study involved:<br><br>• Human participants<br>• Human specimens or tissue<br>• Vertebrate animals or cephalopods<br>• Vertebrate embryos or tissues<br>• Field research<br><br>Write "N/A" if the submission does not require an ethics statement.<br><br>General guidance is provided below. Consult the submission guidelines for detailed instructions. **Make sure that all information entered here is included in the Methods section of the manuscript.**	This study was conducted according to the guidelines laid down in the Declaration of Helsinki and all procedures were approved by the Faculty of Health and Medicine Research Ethics Committee (FHMREC) of Lancaster University, UK (reference FHMREC20128).

**Format for specific study types**<br><br>**Human Subject Research (involving human participants and/or tissue)**<br>• Give the name of the institutional review board or ethics committee that approved the study<br>• Include the approval number and/or a statement indicating approval of this research<br>• Indicate the form of consent obtained (written/oral) or the reason that consent was not obtained (e.g. the data were analyzed anonymously)<br><br>**Animal Research (involving vertebrate animals, embryos or tissues)**<br>• Provide the name of the Institutional Animal Care and Use Committee (IACUC) or other relevant ethics board that reviewed the study protocol, and indicate whether they approved this research or granted a formal waiver of ethical approval<br>• Include an approval number if one was obtained<br>• If the study involved *non-human primates*, add *additional details* about animal welfare and steps taken to ameliorate suffering<br>• If anesthesia, euthanasia, or any kind of animal sacrifice is part of the study, include briefly which substances and/or methods were applied<br><br>**Field Research**<br><br>Include the following details if this study involves the collection of plant, animal, or other materials from a natural setting:<br>• Field permit number<br>• Name of the institution or relevant body that granted permission	
**Data Availability**<br><br>Authors are required to make all data underlying the findings described fully available, without restriction, and from the time of publication. PLOS allows rare exceptions to address legal and ethical concerns. See the PLOS Data Policy and FAQ for detailed information.	Yes - all data are fully available without restriction

A Data Availability Statement describing where the data can be found is required at submission. Your answers to this question constitute the Data Availability Statement and **will be published in the article**, if accepted.<br><br>**Important:** Stating 'data available on request from the author' is not sufficient. If your data are only available upon request, select 'No' for the first question and explain your exceptional situation in the text box.<br><br>Do the authors confirm that all data underlying the findings described in their manuscript are fully available without restriction?	
**Describe where the data may be found in full sentences. If you are copying our sample text, replace any instances of XXX with the appropriate details.**<br><br>• If the data are **held or will be held in a public repository**, include URLs, accession numbers or DOIs. If this information will only be available after acceptance, indicate this by ticking the box below. For example: *All XXX files are available from the XXX database (accession number(s) XXX, XXX.).*<br>• If the data are all contained **within the manuscript and/or Supporting Information files**, enter the following: *All relevant data are within the manuscript and its Supporting Information files.*<br>• If neither of these applies but you are able to provide **details of access elsewhere**, with or without limitations, please do so. For example:<br><br>*Data cannot be shared publicly because of [XXX]. Data are available from the XXX Institutional Data Access / Ethics Committee (contact via XXX) for researchers who meet the criteria for access to confidential data.*<br><br>*The data underlying the results presented in the study are available from (include the name of the third party*	All relevant data are within the manuscript and its Supporting Information files.

and contact information or URL).<br>• This text is appropriate if the data are owned by a third party and authors do not have permission to share the data.<br><br>* typeset	
Additional data availability information:	

Manuscript
Click here to access/download;Manuscript;PlosOne manuscript.docx

Title of article:

The association of diet quality with the mental health of students during their transition to university

Authors and institutions:

Solomis Solomou[1], Heather Robinson[1], Guillermo Perez-Algorta[1]

[1]Faculty of Health and Medicine, Lancaster University, Bailrigg, Lancaster, LA1 4YW, UK.

Corresponding author:

Solomis Solomou

Faculty of Health and Medicine, Lancaster University, Bailrigg, Lancaster, LA14YW, UK.

s.solomou@lancaster.ac.uk

Tel. +447929365396

Short title:

Diet and mental health of university students

Keywords:

Mental Health

Diet Quality

Students

University

Transition

Abbreviations:

Body Mass Index (BMI)

University College London (UCL)

Queen Mary University London (QMUL)

King’s College London (KCL)

Short Form Food Frequency Questionnaire (SFFFQ)

Diet quality score of the SFFFQ (DQS)

International Physical Activity Questionnaire- Short Form (IPAQ)

College Adjustment Questionnaire (CAQ)

Depression Anxiety and Stress Scale (DASS21)

Almost Perfect Scale Revised (APSR)

Statistical Package for the Social Sciences (SPSS)

Unstandardized beta (B)

Standard error for the unstandardized beta (SE B)

Confidence Interval (CI)

Probability value (p)

Correlation coefficient (R)

Title

The association of diet quality with the mental health of students during their transition to university

Abstract

University students are at risk of experiencing mental health and diet quality problems during their transition to university. This study aimed to examine the bidirectional associations between the diet quality and mental health of students during their transition to universities in the UK, and the impact of the transition on diet quality and mental health. The study adopted a cross-sectional design and took place during the first semester of year 2021-2022. Participants were first year undergraduate students at four UK universities, living at student halls. They were invited to participate by completing online surveys. Results showed that a healthy diet was associated with good mental health (b = -3.46, 95% CI [-6.14, -.78]). Unfavourable mental health was associated with having an unhealthy diet (b = -.01, 95% CI [-.02, -.00]). Transition did not moderate the relationship between diet quality and mental health of students, or vice versa (*ps*> .05). Preliminary analyses showed a significant direct effect of good quality of transition to university on good mental health (b = -1.51, 95% CI [-1.88, -1.13]), but did not have an indirect effect via diet quality; there were no significant direct or indirect effects on diet quality, however larger studies are needed to replicate these preliminary analyses. Our current findings can inform university policies and health education research, and further research is needed to determine whether interventions to improve diet quality at the university level could reduce mental health issues, and whether interventions to support students under stress may lead to healthier dietary habits.

Introduction

University students' mental health is a concern and may be negatively affected when transitioning to living on campus (1). Upon entering university, students may experience stress originating from different sources, such as newfound freedom, lack of social support, increased extracurricular workload, elevated academic expectations, and the pressure associated with making vocational choices (2). Other factors like financial difficulties, food insecurity, housing instability, perfectionism, and lack of knowledge to shop and cook healthily can further impact their well-being (3). The difficulties experienced during the transition can lead to the development of mental health issues, such as depression. For example, a meta-analysis of 34 international studies measuring the prevalence of depression in university students (not restricted to first year students) between 1990 and 2010 showed an average prevalence of 30.6% (4). This is an average rate substantially higher than the ~11% prevalence observed in the general population (5).

The transition to university life may also affect diet quality. Students may change their eating habits (6), such as increasing fast food consumption, relying more on take-out meals, and having a reduced intake of fresh food (7,8). These modifications of diet can lead to a phenomenon called "Freshman 15", which refers to gaining 15 lb (6.8 kg) of weight during the first year of university (9). A meta-analysis informed by data from multiple countries reported an average weight gain of 1.4 kg over two terms (1). This weight gain is five times higher compared to changes observed in the general population over a year, and has been observed in at least two thirds of students during their first year of university (10).

The relationships between diet quality and mental health of students, during their transition to universities in the UK, have not been extensively investigated. Moreover, the impact of the quality of the transition on diet and mental health of students at UK universities has not been previously considered by most studies.

This study has aimed to explore the relationships between diet and mental health during the transition to university, by interrogating data in the context of the stress-diathesis model. The stress-diathesis model can facilitate our understanding of how pre-dispositional factors from various domains can cause susceptibility to psychopathology, and eventually lead to conditions that are sufficient for the development of a disorder, such as a mental health disorder (11). In the case of our study, we consider the transition to university to act as the stressor for students that may have a vulnerability to following poor quality diets and/or experiencing mental health difficulties, that could lead to negatives outcomes in terms of diet or mental health.

In addition to considering these relationships in the context of the stress-diathesis model, we also recognise the possibility of the transition to university acting as a direct causal factor on diet quality and mental health. For example, financial difficulties and housing instability during this transition period could cause higher levels of worry and/or rumination (indicators of poor mental health), which could be impacting later on diet quality (12,13).

Our primary research aim was to investigate for any associations of diet quality with the mental health of students and vice versa, during their transition to university, after controlling for gender, perfectionism, exercise, BMI, and university of study. Our secondary aims included investigating whether the quality of the transition moderated the relationship between mental health and diet quality (and vice versa). As part of our secondary research aims, we also investigated whether the transition to university had direct effects on the diet quality and mental health of students. Additionally, we explored whether there were indirect effects of the transition on diet quality through its impact on mental health, as well as on mental health through its influence on diet quality, following a mediational model approach.

Materials and methods

Design

The study adopted a cross-sectional design, taking place during the first semester of the academic year 2021-2022 (from 06-SEP-2021 to 17-DEC-2021). Participants were recruited through convenience and snowballing sampling. This study was conducted according to the guidelines laid down in the Declaration of Helsinki (14) and all procedures were approved by the Faculty of Health and Medicine Research Ethics Committee (FHMREC) of Lancaster University, UK (reference FHMREC20128). Written informed consent was obtained online from all participants.

Participants

Participants were first year undergraduate students at Lancaster University, University College London (UCL), Queen Mary University London (QMUL), and King's College London (KCL) living at student halls (university or private) for the first time. They were included if they were of any gender, ethnicity or race; were at least 18 years old; were first year university students for the first time; were studying at the participating universities only. Participants were excluded if they had been staying at their family home during the week prior to survey completion, and/or if they had been fasting over the week prior to survey completion. Eligible students were invited to complete an online survey hosted in REDCap (a secure web application for building and managing online surveys and databases).

Measures

General questions were used to obtain: 1) the socioeconomic information of the participants (age, gender, relationship status, ethnicity, religion, employment status, and food security); 2) lifestyle information (including smoking status, alcohol use, recreational drug use, type of diet followed, knowledge/skills to cook healthily, and any recent life events); and 3) clinical characteristics (including current weight, weight changes, and height).

The effects of the transition to university were measured by the College Adjustment Questionnaire (CAQ) (15). The questionnaire consists of 14 items, with five items being relevant to educational functioning, five items to relational functioning, and four items to psychological functioning. A general index of adjustment can be computed from these subscales.

The mental health of the participants was monitored by the 21-item depression, anxiety, and stress scale (DASS-21) (16). The DASS-21 is a self-report scale referring to the week preceding the questionnaire completion. The DASS-21 consists of three sections, each of which consists of seven items measuring depression, anxiety and stress respectively (21 items in total). This tool has been

shown to have good construct validity when it was administered to a non-clinical and broadly representative sample of the general adult UK population (17).

Diet quality was measured by the short-form food frequency questionnaire (SFFFQ) (18). The SFFFQ refers to "a typical week over the past month"; for the purposes of this study, participants were asked to complete the questionnaire in relation to the week preceding the questionnaire completion. The SFFFQ is used to provide a diet quality score (DQS), and was developed through expert knowledge about culturally specific foods, in order to tailor the tool to the UK population. The five dietary components considered as indicators of a healthy diet and used to inform the diet quality score (DQS) were fruit intake, vegetable intake, oily fish intake, fat intake and non-milk extrinsic sugar (NMES) intake. Scores of 1–3 are allocated for each of the above components of the DQS, with a score of 3 corresponding to meeting the UK dietary recommendations for that component. The minimum DQS score is 5 and the maximum score (indicating optimum dietary intake) is 15. The SFFFQ consists of 20 items (multiple choice options) and takes approximately fifteen minutes to complete. Scores of 1-5 were considered as indicating poor diet quality, scores 6-10 as moderate diet quality, and 11-15 as good diet quality. The SFFFQ was validated (18) in comparison to a previously validated and comprehensive food frequency questionnaire (FFQ)- a 217 item FFQ created for the UK Women's cohort study (19).

Physical activity was assessed by the short version of international physical activity questionnaire (IPAQ) (20). The IPAQ assesses physical activity over the past week, and has been shown to have good reliability and validity, with criterion validity having a median rho of about 0.30 (21). Perfectionism was assessed by using the Almost Perfect Scale Revised (APSR) (22). The scale consists of 23 items and has been shown to have good reliability and construct validity (23).

Statistical analysis

The association of diet quality with mental health, and mental health with diet quality show effect sizes ranging from small to moderate (14). Hence, in a model with six predictors in total, we aimed to recruit 150 students as minimum to obtain 0.8 power, using an alpha of 0.05. This number of participants was also considered suitable to conduct preliminary analyses regarding our secondary questions, with the aim of informing the development of fully powered future studies.

Exploratory analyses were conducted to evaluate the quality of the data, to identify outliers, and to describe participant characteristics in terms of demographic and clinical information. To respond to the primary research questions, hierarchical regression analyses were conducted, with mental health or diet quality as the dependent variables, and mental health or diet quality, gender, perfectionism, exercise, BMI, and university of study as predictors.

For the moderation analysis we centred our three main variables using sample means (mental health, diet quality and transition to university), including interactive effects accordingly (transition*mental health or transition*diet quality).

Mediation analyses were also performed, following approaches that generate 95% confidence intervals using bootstrapping (24). To run the analyses, the software SPSS (Statistical Package for the Social Sciences) was used (25).

Results

Sample characteristics

The final sample consisted of n=166 students. The sample average age was 19.4 years (SD = 2.9 years), 71% female, of white ethnic background (59%) and "single" (77%). The sample characteristics are summarised in table 1.

In terms of diet (Table 2), most students (68%) reported to be omnivore (i.e. eating various types of food, including meat, vegetables and other animal products), and 76.5% felt they had knowledge and skills to cook healthily. Among respondents, 79% did not experience any food security issues during the 12 months before survey completion while 87% did not report any weight changes since starting university. The mean BMI was 23 kg/m² (SD = 4.9 kg/m²), a result within the healthy adult BMI range of 18.5-24.9 (26).

The mean score of the short form food frequency questionnaire (SFFFQ) was 9.8 (SD = 1.7) out of a maximum score of 15 (Figure 2, panel A), which indicates a moderate-good diet quality. In terms of physical activity, the mean value for metabolic minutes per week was 3590 (SD = 2898) (Figure 2, panel B), which indicates a high level of physical activity. Moreover, 51.8% of the students were under the category of high level of physical activity.

Forty-three percent of the sample reported having experienced stressful events since starting university (Table 3), with financial difficulties being the most frequently reported stressful event. As shown in Table 3, the group that experienced stressful events since starting university exhibited poorer mental health quality compared to the group that did not report stressful events. Overall, this student sample had a reasonably good quality of transition, with lower psychological functioning scores compared to the educational functioning and relational functioning scores (table 3).

Regarding our primary research questions, the results showed that diet quality was a significant predictor of mental health, (*b* = -3.46, 95% CI [-6.14, -.78]). Diet quality explained 4% of the

variability in overall mental health scores, in a model where all predictors together explained 11% of variance (R^2 = .11, F(6, 137)= 2.40, p ≤ .05) (Table 4).

When the model was tested in the opposite direction (research aim 2), mental health was a significant predictor of diet quality (*b* = -.01, 95% CI [-.02, -.00]). Mental health explained 4% of variance on diet quality, in a model that explained 11% in total (R^2 = .11, F(7, 136) = 2.46, p ≤ .05) (Table 5).

Tables 6 and 7 present results for our secondary research questions. With mental health being the dependent variable and all predictors in the model, the interaction effect of the transition and diet quality was not statistically significant (*p* = .27). Diet quality remained as a statistically significant predictor (*b* = -3.13, 95% CI [-5.33, -.93]) and contributed 4% of the total variance explained. Transition scores were also statistically significant (*b* = -1.51, 95% CI [-1.88, -1.13]) and contributed 31% of the variance. Increases on the quality of both diet and transition were associated with reductions in mental health scores.

In a model with diet quality as the dependent variable the interaction effect of the transition and mental health was not statistically significant (*p* = .17). Mental health was a significant predictor (*b* = -.02, 95% CI [-.03, -.00]), explaining 6% of the total variance.

When testing whether the quality of the transition to university had an indirect effect through diet quality on mental health (Figure 1), this indirect effect was not statistically significant (95% bootstrap CI, -.08 to .05). Only the direct effect of the transition to university was found to be statistically different (*t*(161) = -8.66; *p* ≤ .01), meaning that with the level of diet quality being held constant, a better quality of transition by one unit lead to a better mental health score by 1.53 units (Table 8).

In a second model examining whether the quality of the transition to university had an indirect effect through mental health on diet quality (Figure 2), this indirect effect did not reach statistical significance (95% bootstrap CI, -.00 to .04). Additionally, no significant results were observed for the direct effect of transition on diet quality (*p* = .38) (Table 9).

Discussion

This was one of the first studies to investigate the associations between diet quality and mental health of students during the transition to university at UK universities. The study results showed that having a good diet quality during the transition to university was associated with better mental health. These results are in line with the findings of our systematic literature review (27), which found that good diet quality of students was associated with better mental health in terms of depression, anxiety, stress and overall general mental well-being, with the effect sizes being small-moderate. Our results are also in line with previous research with both students transitioning to

university (28) and the general population (29) where better quality diet has been found to be associated with better mental health. A particular parallel is observed with a previous study of students transitioning to a UK-based university, which found that the group with the worst diet quality had higher levels of depression and anxiety compared to groups with better diet quality (28). Previous studies involving the general population (29) have also found that good quality diets were associated with good mental health.

We also found statistically significant differences in the mean diet quality scores depending on diet type, with the pescatarian diet being associated with a statistically significant higher diet quality mean score than the omnivore, vegetarian diet and other (Halal and Kosher) diets. This could imply that the mechanisms responsible for the associations observed in our study could be driven by the presence of elements of Mediterranean diet. It is well known that following healthy diets, like the Mediterranean diet, reduces the risk of depression in the general population (30). These diets reduce inflammation, which could play a contributory role in psychiatric disorders (31,32). The Mediterranean diet is rich in fruits and vegetables, whole grains, nuts and legumes, and olive oil, but also seafood.

Other possible explanations for the observed associations might include vulnerabilities students face during the transition period. For example, the lack of regularity and the loss of home rituals may mean that regular home-made food is replaced by irregular and unhealthy fast-food. In addition, when transitioning to university, students may face financial difficulties. In fact, financial difficulties since starting university was the most frequent stressful event reported by our study participants. Financial difficulties can lead to food insecurity, resulting in students relying more on cheaper unhealthy food- a negative change compared to the food they used to have at home. The effects of these changes could be contributing towards a deterioration of mental health, as students perceive a worsening of their diet and a loss of their usual home dietary routines (33).

Moreover, the fact that students now have to rely on their own cooking means that the quality of the foods they prepare may not be of the same high standard as the food they used to have at home- in cases where they lack the knowledge to cook healthily. Our findings showed that students reporting good knowledge of cooking healthily had better diet quality than those that had no knowledge. There may also be an impact on mental health, as when being unable to cook to the same standard as home food students may experience negative feelings, such as inadequacy and inability to cope by themselves (34).

Our results also showed that there was a statistically significant association of good mental health with good diet quality, however the association was weaker compared to the association of

diet quality with mental health. It might be the case that certain mental health difficulties may be more strongly associated with diet quality than others, for example anxiety and stress (27).

Contrary to expectations based on the stress-diathesis model, our study found no statistically significant moderational effect. This could be attributed to the participants' generally good diet quality and mental health scores, which may have limited variability, making it difficult to detect such effects. Additionally, the study might have lacked the statistical power necessary to identify small effect sizes. Future studies with larger samples are needed to replicate these findings.

Apart from acting as a moderator, we also considered the possibility of transition having a causal effect. So, we ran a mediational model to investigate for any direct and/or indirect effects via diet quality or mental health. We found that quality of the transition to university had a significant direct effect on mental health of students, but not on diet quality. Additionally, our study found no statistically significant indirect effects. The reasons behind the lack of significant findings could be similar to the reasons explaining the lack of significant moderational effects. Specifically, the average diet quality score was within the category of moderate-good diet quality. Larger samples with participants having a broader variability of diet quality may provide further insights. Moreover, studies involving participants with established mental health difficulties may enhance our understanding as to whether the transition has any indirect effects on mental health via diet quality in this student group.

Future studies could also explore new potential moderators and mediators. These include knowledge to cook and shop healthily, food security, weight changes, access to traditional food as moderators, as well as biological mechanisms as candidate mediators. We recommend that future studies use longitudinal designs, enabling researchers to determine the direction of any detected associations. Qualitative studies are also warranted to explore the daily experience of students during this particular period of time.

Our findings have several important implications, including the need to consider strategies at the university level to raise awareness of the associations between diet and mental health. The findings also highlight the need for universities to review how they operate in order to promote better diet quality and mental health of students during their university experience, and especially during the transition to university.

Considering the fact that food insecurity may be a factor affecting the diet quality of students, food assistance services may be helpful for food insecure students (3). The quality of food offered as part of food assistance would also be relevant, as diet quality may only improve if fresh and healthy options are offered as part of the food assistance, rather than options that may be low in terms of nutritional quality (35).

The study findings suggest that good knowledge to cook healthily is associated with a better diet quality of students. This means that there may be scope to target and improve the food literacy of university students; this may increase the confidence of students to cook healthily by themselves rather than relying on take away foods of low nutritional value, thus leading to a better diet quality. Food literacy would not just include cooking skills, but also education relevant to planning, managing, budgeting, selecting and preparing food (3,36). Interventions that could be considered include nutrition education lectures, cooking demonstrations, and discussion (37), individualised dietary counselling meetings with a clinical nutritionist (38), dietary advice via newsletters (39).

In terms of interventions targeting the mental health of students, these could include traditional counselling and technology-delivered psychological interventions, (including online cognitive behavioural therapy) (40), mindfulness-based interventions (41), educational/personalised mail feedback interventions to provide students with feedback about their symptoms (42), recreation programmes (such as meditation and exercise), as well as psychoeducation interventions to provide information relevant to stress, coping, and ways to relax (43).

Our study did not collect information about the food environment and food purchasing at the university campuses, as this was not the study scope. However, previous studies have reported that a higher frequency of food and beverage purchasing on campuses was associated with the consumption of energy-dense and nutrient-poor foods (44,45). There may be scope for relevant university policies to be developed. For example, universities may need to review what economic and planning decisions they make in terms of which franchises they allow onto campuses, and influence what is sold in the university shops. Additionally, there could be consideration for exclusion zones around the university campuses for franchises and shops that offer unhealthy foods. Development of such policies would allow monitoring the types of food stores allowed on campuses, as well as around campuses; this may limit the availability of fast foods and maximise the availability of healthy options at cheaper prices (46).

There are a number of strengths to this study. For example, the study had a strong theoretical foundation; it did not adopt a narrow biomedical approach, and instead used a stress diathesis model of biopsychosocial factors to investigate and analyse exposure and outcomes, taking into consideration relevant confounding variables. The study consisted of more participants (n=166) than the minimum necessary (n=150), meaning it was adequately powered to answer the primary aims.

There were also some limitations to this study. For example, the study was cross-sectional therefore the directionality of effects could not be determined. The study only addressed depression, anxiety and stress, however there are other mental health issues such as eating disorders that often develop around the age of moving to university. Hence, we cannot rule out the possibility that some

students with eating disorders may have taken part in our study. Another issue that was not addressed by our study is that relationships between diet quality and mental health during the transition to university could also be partly explained by biological mechanisms, such as inflammation and dysregulated hypothalamic pituitary adrenal (HPA) axis.

This study has filled a research gap and contributed to our knowledge of the associations between the diet quality and mental health of students during their transition to universities in the UK. The findings can act as a reference for policy makers in terms of designing future university policies relevant to the mental health of students, the diet quality of students, the health education of students, the food security of students and the university food environment. The findings inform the design of relevant studies in the future that may involve clinical samples, and highlight the need for further research including qualitative studies, as well as intervention studies to determine whether interventions to improve diet quality at the university level could reduce mental health issues, and whether interventions to support students under stress may lead to healthier dietary habits.

Acknowledgements

The present study did not receive any funding.

The authors have no financial or personal conflicts of interest to declare.

The authors’ contributions are as follows: SS and GPA contributed to the study design, data collection and data analyses; SS was the principal investigator and contributed to the study design and interpretation of the findings and wrote the manuscript; GPA and HR contributed to the data analyses and interpretation. All authors read and approved the final version of the manuscript.

References

1. Vadeboncoeur C, Townsend N, Foster C. A meta-analysis of weight gain in first year university students: is freshman 15 a myth? BMC Obes. 2015 May 28;2:22.
2. Schwartz SJ, Côté JE, Arnett JJ. Identity and agency in emerging adulthood. Youth Soc. 2005 Dec;37(2):201–229.
3. Shi Y, Grech A, Allman-Farinelli M. Diet Quality among Students Attending an Australian University Is Compromised by Food Insecurity and Less Frequent Intake of Home Cooked Meals. A Cross-Sectional Survey Using the Validated Healthy Eating Index for Australian Adults (HEIFA-2013). Nutrients. 2022 Oct 27;14(21).
4. Ibrahim AK, Kelly SJ, Adams CE, Glazebrook C. A systematic review of studies of depression prevalence in university students. J Psychiatr Res. 2013 Mar;47(3):391–400.
5. Lim GY, Tam WW, Lu Y, Ho CS, Zhang MW, Ho RC. Prevalence of Depression in the Community from 30 Countries between 1994 and 2014. Sci Rep. 2018 Feb 12;8(1):2861.

6. de Vos P, Hanck C, Neisingh M, Prak D, Groen H, Faas MM. Weight gain in freshman college students and perceived health. Prev Med Rep. 2015 Apr 1;2:229–234.
7. Graham MA, Jones AL. Freshman 15: valid theory or harmful myth? J Am Coll Health. 2002 Jan;50(4):171–173.
8. Lowry R, Galuska DA, Fulton JE, Wechsler H, Kann L, Collins JL. Physical activity, food choice, and weight management goals and practices among U.S. college students. Am J Prev Med. 2000 Jan;18(1):18–27.
9. Brown C. The information trail of the "Freshman 15"--a systematic review of a health myth within the research and popular literature. Health Info Libr J. 2008 Mar;25(1):1–12.
10. Levitsky DA, Halbmaier CA, Mrdjenovic G. The freshman weight gain: a model for the study of the epidemic of obesity. Int J Obes Relat Metab Disord. 2004 Nov;28(11):1435–1442.
11. Ingram RE, Luxton DD. Vulnerability-Stress Models. Development of Psychopathology: A Vulnerability-Stress Perspective. 2455 Teller Road, Thousand Oaks California 91320 United States : SAGE Publications, Inc.; 2005. p. 32–46.
12. Archuleta KL, Dale A, Spann SM. College Students and Financial Distress: Exploring Debt, Financial Satisfaction, and Financial Anxiety. Journal of Financial Counseling and Planning. 2013;
13. Kim Y, Murphy J. Mental Health, Food Insecurity, and Economic Hardship among College Students during the COVID-19 Pandemic. Health Soc Work. 2023 Mar 10;
14. World Medical Association. World Medical Association Declaration of Helsinki: ethical principles for medical research involving human subjects. JAMA. 2013 Nov 27;310(20):2191–2194.
15. O'Donnell MB, Shirley LA, Park SS, Nolen JP, Gibbons AM, Rosén LA. The college adjustment questionnaire: A measure of students' educational, relational, and psychological adjustment to the college environment. J Coll Stud Dev. 2018;59(1):116–121.
16. Lovibond PF, Lovibond SH. The structure of negative emotional states: comparison of the Depression Anxiety Stress Scales (DASS) with the Beck Depression and Anxiety Inventories. Behav Res Ther. 1995 Mar;33(3):335–343.
17. Henry JD, Crawford JR. The short-form version of the Depression Anxiety Stress Scales (DASS-21): construct validity and normative data in a large non-clinical sample. Br J Clin Psychol. 2005 Jun;44(Pt 2):227–239.
18. Cleghorn CL, Harrison RA, Ransley JK, Wilkinson S, Thomas J, Cade JE. Can a dietary quality score derived from a short-form FFQ assess dietary quality in UK adult population surveys? Public Health Nutr. 2016 Nov;19(16):2915–2923.
19. Cade JE, Burley VJ, Greenwood DC, UK Women's Cohort Study Steering Group. The UK Women's Cohort Study: comparison of vegetarians, fish-eaters and meat-eaters. Public Health Nutr. 2004 Oct;7(7):871–878.
20. Booth M. Assessment of physical activity: an international perspective. Res Q Exerc Sport. 2000 Jun;71 Suppl 2:114–120.
21. Craig CL, Marshall AL, Sjöström M, Bauman AE, Booth ML, Ainsworth BE, et al. International physical activity questionnaire: 12-country reliability and validity. Med Sci Sports Exerc. 2003 Aug;35(8):1381–1395.
22. Rice KG, Ashby JS. An efficient method for classifying perfectionists. J Couns Psychol. 2007 Jan;54(1):72–85.
23. Slaney RB, Rice KG, Mobley M, Trippi J, Ashby JS. The revised almost perfect scale. Measurement and Evaluation in Counseling and Development. 2001 Oct 1;34(3):130–145.

24. Preacher KJ, Hayes AF. SPSS and SAS procedures for estimating indirect effects in simple mediation models. Behav Res Methods Instrum Comput. 2004 Nov;36(4):717–731.
25. IBM Corp. IBM SPSS Statistics for Windows. Armonk, NY: IBM Corp; 2021.
26. NIH. Managing Overweight and Obesity in Adults: Systematic Evidence Review from the Obesity Expert Panel | NHLBI, NIH [Internet]. 2013 [cited 2022 Apr 26]. Available from: https://www.nhlbi.nih.gov/health-topics/managing-overweight-obesity-in-adults
27. Solomou S, Logue J, Reilly S, Perez-Algorta G. A systematic review of the association of diet quality with the mental health of university students: implications in health education practice. Health Educ Res. 2023 Jan 20;38(1):28–68.
28. Romijn AR. Relationships among eating behaviour, diet quality, mental health and wellbeing in undergraduate students transitioning to university. ISNPR. 2019 Oct 21;
29. Sánchez-Villegas A, Henríquez-Sánchez P, Ruiz-Canela M, Lahortiga F, Molero P, Toledo E, et al. A longitudinal analysis of diet quality scores and the risk of incident depression in the SUN Project. BMC Med. 2015 Sep 17;13:197.
30. Sánchez-Villegas A, Delgado-Rodríguez M, Alonso A, Schlatter J, Lahortiga F, Serra Majem L, et al. Association of the Mediterranean dietary pattern with the incidence of depression: the Seguimiento Universidad de Navarra/University of Navarra follow-up (SUN) cohort. Arch Gen Psychiatry. 2009 Oct;66(10):1090–1098.
31. Bauer ME, Teixeira AL. Inflammation in psychiatric disorders: what comes first? Ann N Y Acad Sci. 2019 Feb;1437(1):57–67.
32. Osimo EF, Cardinal RN, Jones PB, Khandaker GM. Prevalence and correlates of low-grade systemic inflammation in adult psychiatric inpatients: An electronic health record-based study. Psychoneuroendocrinology. 2018 May;91:226–234.
33. Bauch C, Torheim LE, Almendingen K, Molin M, Terragni L. Food Habits and Forms of Food Insecurity among International University Students in Oslo: A Qualitative Study. Int J Environ Res Public Health. 2023 Feb 2;20(3).
34. Hyytinen H, Tuononen T, Nevgi A, Toom A. The first-year students' motives for attending university studies and study-related burnout in relation to academic achievement. Learn Individ Differ. 2022 Jul;97:102165.
35. Efrati Philip D, Baransi G, Shahar DR, Troen AM. Food-Aid Quality Correlates Positively With Diet Quality of Food Pantry Users in the Leket Israel Food Bank Collaborative. Front Nutr. 2018 Dec 18;5:123.
36. Vidgen HA, Gallegos D. Defining food literacy and its components. Appetite. 2014 May;76:50–59.
37. Agarwal U, Mishra S, Xu J, Levin S, Gonzales J, Barnard ND. A multicenter randomized controlled trial of a nutrition intervention program in a multiethnic adult population in the corporate setting reduces depression and anxiety and improves quality of life: the GEICO study. Am J Health Promot. 2015 Apr;29(4):245–254.
38. Einvik G, Ekeberg O, Lavik JG, Ellingsen I, Klemsdal TO, Hjerkinn EM. The influence of long-term awareness of hyperlipidemia and of 3 years of dietary counseling on depression, anxiety, and quality of life. J Psychosom Res. 2010 Jun 1;68(6):567–572.
39. Jenkinson CM, Doherty M, Avery AJ, Read A, Taylor MA, Sach TH, et al. Effects of dietary intervention and quadriceps strengthening exercises on pain and function in overweight people with knee pain: randomised controlled trial. BMJ. 2009 Aug 18;339:b3170.

40. Farrer L, Gulliver A, Chan JKY, Batterham PJ, Reynolds J, Calear A, et al. Technology-based interventions for mental health in tertiary students: systematic review. J Med Internet Res. 2013 May 27;15(5):e101.
41. Bamber MD, Kraenzle Schneider J. Mindfulness-based meditation to decrease stress and anxiety in college students: A narrative synthesis of the research. Educational Research Review. 2016 May;18(18):1–32.
42. Reavley N, Jorm AF. Prevention and early intervention to improve mental health in higher education students: a review. Early Interv Psychiatry. 2010 May;4(2):132–142.
43. Conley CS, Durlak JA, Dickson DA. An evaluative review of outcome research on universal mental health promotion and prevention programs for higher education students. J Am Coll Health. 2013;61(5):286–301.
44. Roy R, Rangan A, Hebden L, Yu Louie JC, Tang LM, Kay J, et al. Dietary contribution of foods and beverages sold within a university campus and its effect on diet quality of young adults. Nutrition. 2017 Feb;34:118–123.
45. Whatnall MC, Soo ZM, Patterson AJ, Hutchesson MJ. University Students Purchasing Food on Campus More Frequently Consume More Energy-Dense, Nutrient-Poor Foods: A Cross-Sectional Survey. Nutrients. 2021 Mar 24;13(4).
46. Shi Y, Wang Q, Norman C, Allman-Farinelli M, Colagiuri S. It is time to make policy for healthier food environments in australian universities. Nutrients. 2018 Dec 4;10(12).

Table 1. Demographic information

University College London (UCL), Queen Mary University London (QMUL), King’s College London (KCL)

**Variables**	
Age (years)	
n (missing n)	162 (4)
Mean (sd)	19.4 (2.9)
Range	18-41
Gender, *n (%)*	
Female	118 (71)
Male	44 (27)
Other	4 (2)
University, *n (%)*	
Lancaster University	55 (33)
UCL	13 (8)
QMUL	57 (34)
KCL	37 (22)
Not known	4 (2)
University Location, [illegible]	
Urban (London)	107 (65)
Rural (Lancaster)	55 (31)
Not known	4 (2)
Ethnic background, n (%)	
White	98 (59)
Asian or Asian British	24 (15)
Chinese	14 (8)
Other (Black, mixed, other ethnic group)	30 (18)
Relationship status, n (%)	
Single	127 (77)
In a relationship	35 (21)
Other	4 (2)
Student status, n (%)	
Home Student	115 (69)

Overseas Student	50 (30)
Not known	1 (1)
Smoking status, n (%)	
Current smoker	19 (11)
Ex-smoker	5 (3)
Never smoked > 100 cigarettes	142 (86)
Alcohol consumption (last week), n (%)	
Rarely/no alcohol	82 (49)
<14 units	55 (33)
14-21 units	21 (13)
>21 units	8 (5)
Use of recreational drugs, n (%)	
Yes	11 (7)
No	155 (93)

Table 2. Information about diet, weight and physical activity

Body Mass Index (BMI).

**Variables**	
Food insecure (last 12 months), n (%)	
Often	5 (3)
Sometimes	30 (18)
Never	131 (79)
Type of diet followed, n (%)	
Omnivore	113 (68)
Vegetarian	29 (18)
Vegan	5 (3)
Pescatarian	7 (4)
Other (Halal, Kosher)	12 (7)
Subjective knowledge/skills for healthy cooking n (%)	
Yes	127 (76.5)
No	39 (23.5)
Weight (kg)	
n (missing n)	162 (4)
Mean (sd)	65.1 (15.7)
Range	39.1-131.0

Weight change since starting university	
No change	
n (%)	87 (52)
Weight gain (kg)	
n (%)	27 (16)
mean (sd)	3.8 (3.0)
Weight loss (kg)	
n (%)	50 (30)
mean (sd)	3.4 (3.6)
Not known	
n (%)	2 (1)
BMI (kg/m²)	
n (missing n)	156 (10)
Mean (sd)	23.0 (4.9)
Range	14.7-41.0
Diet quality score (SFFFQ)	
n (missing n)	166 (0)
Mean (sd)	9.8 (1.7)
Range	5-14
Physical activity n (%)	
Low	13 (7.8)
Moderate	61 (36.7)
High	86 (51.8)
Not known	6 (3.6)
Metabolic minutes per week	
n (missing n)	160 (6)
Mean (sd)	3590 (2898)
Range	0-14238

Panel A. Distribution of the diet quality scores of the SFFFQ questionnaire

Panel B. Distribution of the metabolic minutes per week of the IPAQ questionnaire

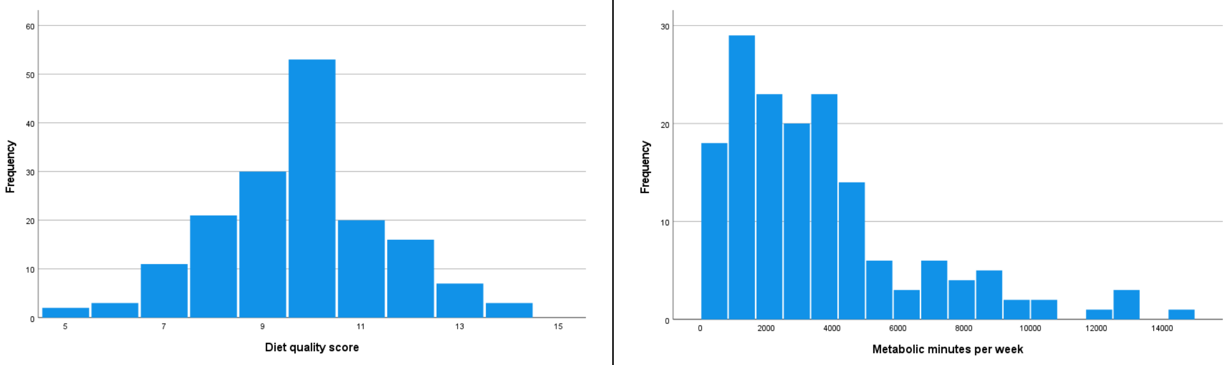

Figure 1. Diet quality and exercise score distributions

Short Form Food Frequency Questionnaire (SFFFQ), Diet quality score of the SFFFQ (DQS), International Physical Activity Questionnaire- Short Form (IPAQ).

Table 3. Information about mental health and adjustment to university

College Adjustment Questionnaire (CAQ), Depression Anxiety and Stress Scale (DASS21), Almost Perfect Scale Revised (APSR).

**Demographics/variables**	**All**	**Stressful events**	**No stressful events**	**t-test**
Stressful life events since starting university n (%)	166 (100)	72 (43)	94 (57)	N/A
Adjustment to university score (CAQ)				
Educational functioning				
Mean (sd)	17.7 (3.7)	17.4 (4.0)	18.0 (3.4)	t(164) = 1.062,
Range	5-25	5-25	9-25	p = 0.290
Relational functioning				
Mean (sd)	15.5 (5.3)	14.8 (5.3)	15.9 (5.3)	t(164) = 1.327,
Range	5-25	5-25	5-25	p = 0.186
Psychological functioning				
Mean (sd)	13.4 (4.1)	11.9 (4.0)	14.5 (3.7)	t(164) = 4.387,
Range	4-20	5-20	4-20	p < 0.001
Mental health score (DASS21)				
Depression				

Mean (sd)<br>*Range*	14.7 (11.5)<br>0-42	17.3 (11.4)<br>0-42	12.7 (11.2)<br>0-42	t(164) = 2.586, p = 0.011
Anxiety<br>*Mean (sd)*<br>*Range*	13.3 (10.1)<br>0-42	16.9 (9.9)<br>0-42	10.5 (9.5)<br>0-42	t(164) = 4.219, p < 0.001
Stress<br>*Mean (sd)*<br>*Range*	15.8 (10.7)<br>0-42	19.6 (10.7)<br>0-42	12.9 (9.9)<br>0-40	t(164) = 4.184, p < 0.001
Total DASS21 score<br>*Mean (sd)*<br>*Range*	43.8 (28.7)<br>0-126	53.8 (27.8)<br>0-126	36.1 (27.1)<br>0-118	t(164) = 4.115, p < 0.001
Perfectionism score (APSR)<br>*n (missing n)*<br>*Mean (sd)*<br>*Range*	162 (4)<br>32.0 (7.4)<br>9-42	71 (1)<br>32.8 (7.7)	91 (3)<br>31.3 (7.1)	t(160) = 1.230<br>p = 0.220

Panel A. Distribution of the depression scores of the DASS21 questionnaire

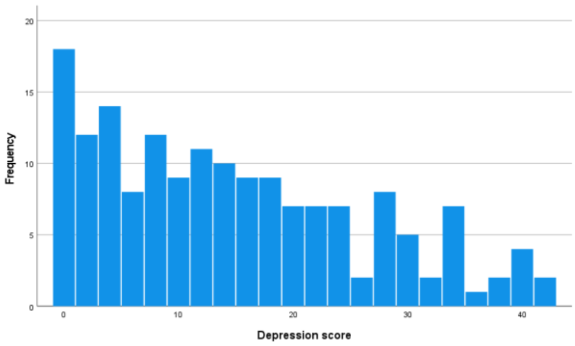

Panel B. Distribution of the anxiety scores of the DASS21 questionnaire

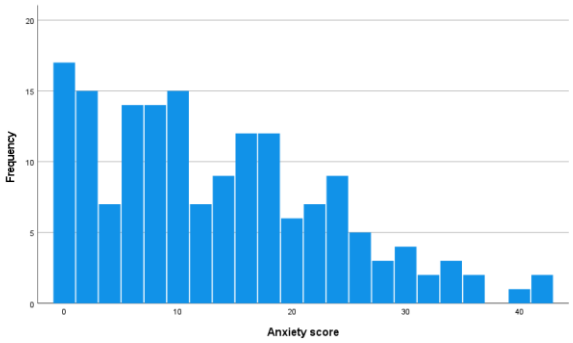

Panel C. Distribution of the stress scores of the DASS21 questionnaire

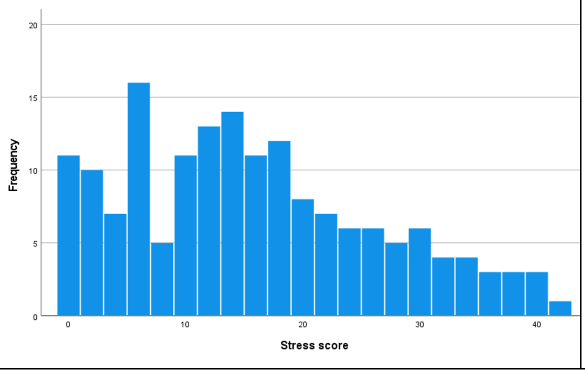

Panel D. Distribution of the total scores of the DASS21 questionnaire

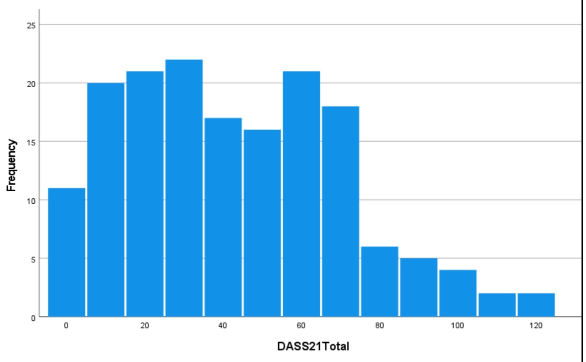

Figure 2. Distribution of the depression, anxiety, stress and total scores of the DASS21 questionnaire. Depression Anxiety and Stress Scale (DASS21)

Panel A. Distribution of the educational functioning scores of the CAQ questionnaire

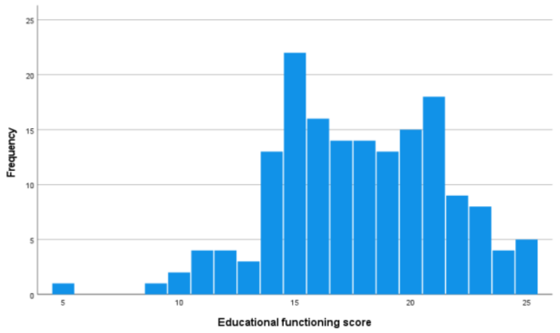

Panel B. Distribution of the relational functioning scores of the CAQ questionnaire

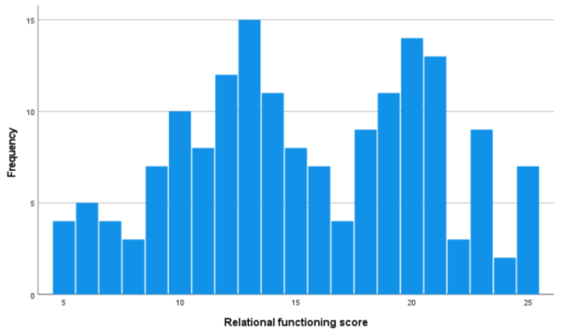

Panel C. Distribution of the psychological functioning scores of the CAQ questionnaire

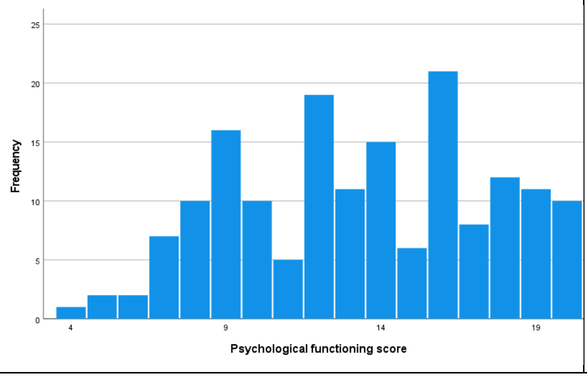

Panel D. Distribution of the standards scores of the APSR questionnaire

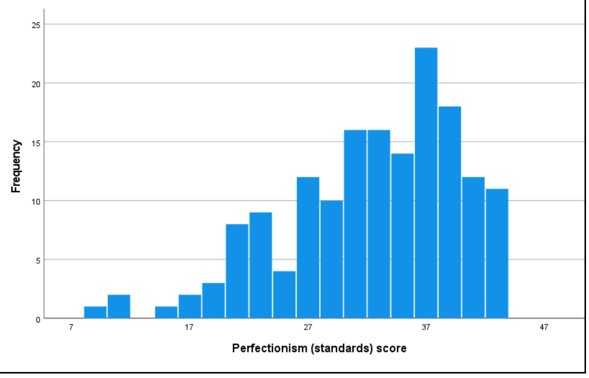

Figure 3. Distribution of the educational, relational and psychological functioning scores of the CAQ questionnaire, and of the standards scores of the APSR questionnaire. College Adjustment Questionnaire (CAQ), Almost Perfect Scale Revised (APSR)

Table 4. Sequential regression of variables (including diet quality scores) on the overall mental health (DASS-21)

**Variables**	**DASS21 score**	**Gender 0 (female)**	**Gender 1 (male)**	**Gender 2 (other)**	**Perfecti -onism**	**Exercise**	**BMI**	**University Location**	**DQS score**	**B**	**SE B**	**95% CI for B**	**sr^2 (incremental)**
Gender 0 (female)	.14*												
Gender 1 (male)	-.20**	-.93**								-12.80	5.50	-23.66, -1.93	
Gender 2 (other)	.15*	-.26**	-.10							17.02	14.07	-10.81, 44.84	.06*
Perfectionism (APSR)	.15*	.14*	-.20**	.13						.37	.31	-.25, .99	.01
Exercise	-.02	-.16*	.14*	.07	.05					.00	.00	-.00, .00	.00
BMI	.01	-.09	.10	-.02	-.02	-.10				.13	.48	-.81, 1.07	.01
University Location	.03	.23**	-.21**	-.07	.08	-.20**	-.06			.37	5.12	-9.75, 10.49	.00
DQS score	-.19**	.15*	-.13	-.07	.01	.18*	-.07	.06		-3.46	1.35	-6.14, -.78	.04**
Intercept										64.60	20.63		
Means	44.24	.71	.26	.03	31.48	3353.69	23.06	.69	9.73				
Standard deviations	28.06	.46	.44	.16	7.46	2691.68	4.85	.47	1.74				
													R^2 = .11 Adjusted R^2= .06 R = .33

Depression Anxiety and Stress Scale (DASS21), Almost Perfect Scale Revised (APSR), Diet Quality Score of the Short Form Food Frequency Questionnaire (DQS)
Unstandardized beta (B), standard error for the unstandardized beta (SE B), CI (confidence interval), probability value (p), correlation coefficient (R)
*p ≤ .05, **p ≤ .01

Table 5. Sequential regression of variables (including overall mental health scores) on the diet quality (DQS) score

Variables	DQS score	Gender 0 (female)	Gender 1 (male)	Gender 2 (other)	Perfecti-onism	Exercise	BMI	University Location	DASS21 score	B	SE B	95% CI for B	sr^2 (incremental)
Gender 0 (female)	.15*												
Gender 1 (male)	-.13	-.93**								-.75*	.34	-1.42, -.07	
Gender 2 (other)	-.07	-.26**	-.10							-.71	.87	-2.44, 1.01	.02
Perfectionism (APSR)	.01	.14*	-.20**	.13						-.00	.02	-.04, .04	.00
Exercise	.18*	-.16*	.14*	.07	.05					.00	.00	.00, .00	.04**
BMI	-.07	-.09	.10	-.02	-.02	-.10				-.01	.03	-.07, .05	.00
University Location	.06	.23**	-.21**	-.07	.08	-.20**	-.06			.24	.32	-.39, .86	.00
DASS21 score	-.19*	.14*	-.20**	.15*	.15*	-.02	.01	.03		-.01	.01	-.02, -.00	.04**
Intercept										10.15	1.00		
Means	9.73	.71	.26	.03	31.48	3353.69	23.06	.69	44.24				
Standard deviations	1.74	.46	.44	.16	7.46	2691.68	4.85	.47	28.06				
													R^2 = .11 Adjusted R^2 = .07, R = .34*

Depression Anxiety and Stress Scale (DASS21), Almost Perfect Scale Revised (APSR), Diet Quality Score of the Short Form Food Frequency Questionnaire (DQS)

Unstandardized beta (B), standard error for the unstandardized beta (SE B), CI (confidence interval), probability value (p), correlation coefficient (R)
*p ≤ .05, **p ≤ .01

Table 6. Sequential regression of variables (including multiplicative interactive effects of transition and diet quality) on the overall mental health (DASS-21)

Variables	B	SE B	95% CI for B	β	sr^2 (incremental)
Gender 0 (female)					
Gender 1 (male)	-6.73	4.55	-15.73, 2.27	-.11	
Gender 2 (other)	13.35	11.48	-9.35, 36.05	.08	.06*
Perfectionism (APSR)	.51	.26	-.00, 1.01	.13	.01
Exercise	.00	.00	.00, .00	.13	.00
BMI	-.20	.39	-.98, .57	-.04	.00
University Location	-.85	4.19	-9.14, 7.44	-.01	.00
CAQ score	-1.51**	.19	-1.88, -1.13	-.56	.31**
DQS score	-3.13**	1.11	-5.33, -.93	-.19	.04**
CAQ*DQS	.13	.12	-.10, .36	.08	.01
					R^2 = .42<br>Adjusted R^2 = .38<br>R = .65**

Depression Anxiety and Stress Scale (DASS21), Almost Perfect Scale Revised (APSR), College Adjustment Questionnaire (CAQ), Diet Quality Score of the Short Form Food Frequency Questionnaire (DQS), CAQ*DQS multiplicative effect of transition and diet quality
Unstandardized beta (B), standard error for the unstandardized beta (SE B), Confidence Intervals (CI), standardized beta (β), probability value (p), correlation coefficient (R)
*p ≤ .05, **p ≤ .01

Table 7 . Sequential regression of variables ((including multiplicative interactive effects of transition and mental health) on the diet quality (DQS) score

Variables	B	SE B	95% CI for B	β	sr^2 (incremental)
Gender 0 (female)					
Gender 1 (male)	-.64	.34	-1.31, .041	-.16	
Gender 2 (other)	-.58	.87	-2.30, 1.14	-.06	.02
Perfectionism (APSR)	.00	.02	-.04, .04	.01	.00
Exercise	.00	.00	.00, .00	.24	.04*
BMI	-.01	.03	-.07, .04	-.04	.00
University Location	.18	.32	-.45, .80	.05	.00
CAQ score	-.02	.02	-.06, .01	-.15	.00
DASS-21 score	-.02**	.01	-.03, -.00	-.30	.06**
CAQ*DASS-21	.00	.00	.00, .00	.11	.01
					R^2 = .14<br>Adjusted R^2 = .08<br>R = .37*

Depression Anxiety and Stress Scale (DASS21), Almost Perfect Scale Revised (APSR), College Adjustment Questionnaire (CAQ), Diet Quality Score of the Short Form Food Frequency Questionnaire (DQS), CAQ*DASS-21 multiplicative effect of transition and mental health
Unstandardized beta (B), standard error for the unstandardized beta (SE B), confidence intervals (CI), standardized beta (β), probability value (p), correlation coefficient (R)
*p ≤ .05, **p ≤ .01

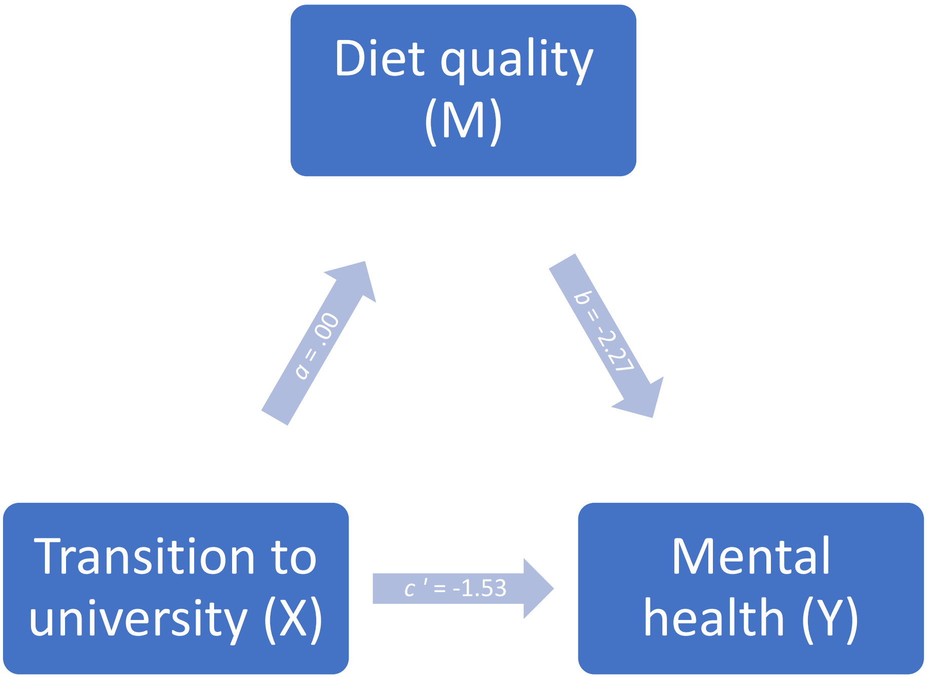

Figure 4. Simple mediation model for the influence of the transition to university and diet quality on the mental health of university students, in the form of a statistical diagram

Table 8. Model Coefficients for the influence of transition to university on mental health

Standard deviation (SE), Probability value (p), Coefficient (coeff.), correlation coefficient (R), Fvalue (F)

		Consequent						
		M (Diet quality)				*Y* (Mental health)		
Antecedent		Coeff.	*SE*	*p*		Coeff.	*SE*	*p*
X (Transition)	*a*	.00	.01	.72	*c'*	-1.53	.18	< .001
M (Diet quality)		—	—	—	*b*	-2.27	1.06	< .05
constant	*iM*	9.53	.62	< .001	*iY*	137.33	13.19	< .001
			$R^2 = .00$				$R^2 = .33$	
		$F(1, 164) = .13, p = .72$				$F(2, 163) = 40.29, p < .001$		

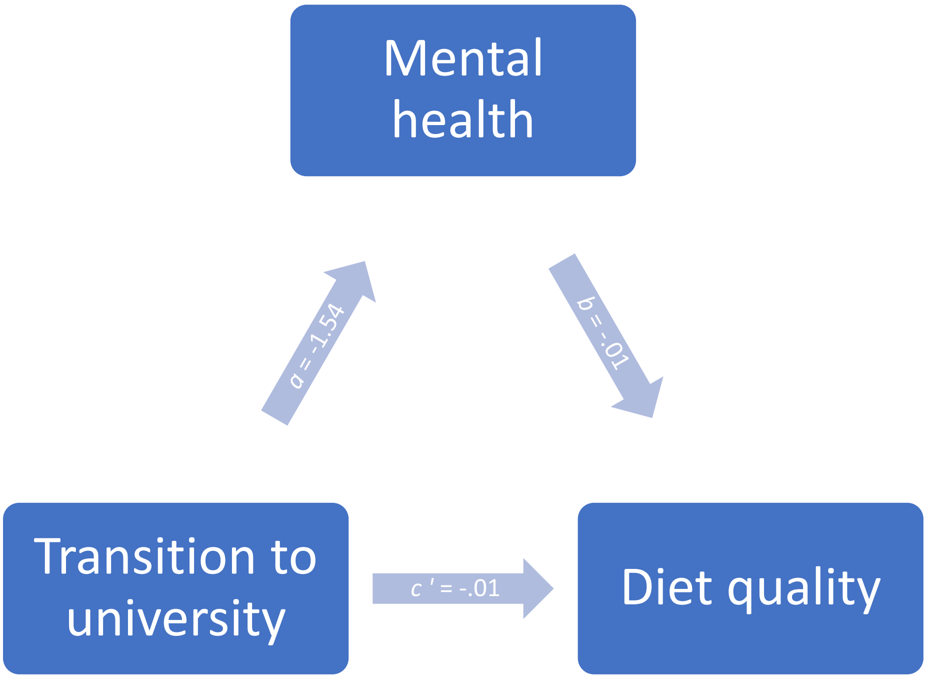

Figure 5. Simple mediation model for the influence of the transition to university and mental health on the diet quality of university students, in the form of a statistical diagram

Table 9. Model Coefficients for the influence of transition to university on diet quality

Standard deviation (SE), Probability value (p), Coefficient (coeff.), correlation coefficient (R), Fvalue (F)

	Consequent							
		M (Mental health)				*Y* (Diet quality)		
Antecedent		Coeff.	*SE*	*p*		Coeff.	*SE*	*p*
X (Transition)	*a*	-1.54	.18	< .001	*c*′	-.01	.02	.38
M (Mental health)		—	—	—	*b*	-.01	.01	< .05
constant	*iM*	115.74	8.55	< .001	*iY*	10.91	.89	< .001
			R^2 = .31				R^2 = .03	
		F(1, 164) = 74.42, p = < .001				F(2, 163) = 2.34, p = .10		
