# Supplementary material for: The association of diet quality with the mental health of students during their transition to university
Source: PLoS One. 2024 Oct 15;19(10):e0312123. doi: 10.1371/journal.pone.0312123 (PMC11478860; doi:10.1371/journal.pone.0312123)
Supplement: Attachment — Submitted filename: Rebuttal letter Response to reviewers.docx [file pone.0312123.s002.docx]

| **Dear reviewers,**  **Thank you for your constructive feedback. Please find below table with action on requested changes.** |  |  |
| --- | --- | --- |
| **Change requested** | **Action** | **Page** |
| Journal requirements |  |  |
| 1. Please ensure that your manuscript meets PLOS ONE's style requirements | Manuscript meets requirements |  |
| 2. Please confirm at this time whether or not your submission contains all raw data required to replicate the results of your study. If your submission does not contain these data, please either upload them as Supporting Information files or deposit them to a stable, public repository and provide us with the relevant URLs, DOIs, or accession numbers. | Submission contains raw data |  |
| *Additional editor comments* |  |  |
| General comments |  |  |
| 1. The cross-sectional nature of your study limits the ability to draw causal inferences between diet quality and mental health. We recommend adding a more explicit discussion of this limitation in both the abstract and the discussion sections. Additionally, consider suggesting that future research should employ longitudinal designs to better capture the dynamics of these relationships over time.   Action: Please revise the manuscript to clearly articulate this limitation and suggest directions for future research. | The limitations of the cross-sectional nature of the study, as well as suggestions for designing longitudinal studies in the future have been discussed further both in abstract and discussion section. | 1, 12 |
| Sample Size and Generalizability |  |  |
| 1. While your sample size meets the minimum requirement for your primary analyses, it may be insufficient to detect smaller effects, particularly in the moderation and mediation analyses. Furthermore, the study’s focus on students from four UK universities may limit the generalizability of your findings.   Action: We suggest that you address these limitations in the discussion, emphasizing the need for larger and more diverse samples in future studies. | The limitations of the sample size, the limitations of generalisability of the results, and relevant recommendations for future studies have been added to the discussion. | 12 |
| Effect Size and Practical Significance |  |  |
| 1. The effect sizes reported in your study are small, which raises questions about their practical significance. It is important to present these findings with an appropriate level of caution. Action: Please revise the discussion to emphasize the small effect sizes and their implications, ensuring that readers understand the modest nature of these associations. | The fact that the effect sizes were small has been further discussed | 8-9 |
| Overinterpretation of Non-Significant Findings |  |  |
| 1. The manuscript discusses non-significant findings from the moderation and mediation analyses at length. While it is important to report these results, care should be taken not to overinterpret them. Action: We recommend revising these sections to clearly state that these findings are non-significant and to avoid speculative interpretations. Focus on the limitations that may have contributed to these results, such as sample size and variability. | Revisions have been made, focusing on small sample size and limited variability as the main limitations. | 10 |
| Reliance on Self-Reported Data: |  |  |
| 1. The study relies on self-reported measures for both diet quality and mental health, which can introduce biases. While self-reports are common in this type of research, it is important to acknowledge the limitations associated with this approach. Action: Please include a discussion of the potential biases associated with self-reported data and suggest ways that future research could incorporate more objective measures. | Discussion of limitations of self reported measures has been added, as well as future recommendations. | 12-13 |
| Biological Mechanisms Discussion |  |  |
| 1. The manuscript speculates about biological mechanisms (e.g., inflammation, HPA axis dysregulation) that might link diet and mental health, but these are not directly measured in the study. Action: We suggest that you revise this section to clarify that these are hypotheses based on existing literature and not findings from your study. Consider recommending that future studies include biological measures to explore these mechanisms further. | The section has been revised, and relevant future recommendations made | 12 |
| Ethical considerations |  |  |
| 1. While you mention that ethical approval was obtained, it would be beneficial to provide more detail about how participant privacy and data security were protected, particularly given the sensitive nature of mental health data. Action: Please expand the ethics statement in the methods section to include details on how data confidentiality was maintained and how participant privacy was ensured. | A relevant ethics statement relevant to data anonymity and confidentiality has been added. | 5 |
| Tables |  |  |
| 1. There are few issues with the tables, including inconsistent use of symbols for significance levels, lack of units for variables like BMI, and inadequate explanations for certain terms (e.g., sr²). Additionally, the R² and Adjusted R² values are reported without interpretation. Action: We recommend revising the tables to ensure consistency and clarity. Please provide units where applicable, define all terms in the legend, and offer brief interpretations of the R² and Adjusted R² values. | Tables and their legends have been updated and amended as advised | 18-32 |
| Justification of the Study |  |  |
| 1. While the introduction provides a general background on the relationship between diet and mental health, it could benefit from a stronger justification for why this specific study is needed, particularly in the context of UK universities. Action: Consider expanding the introduction to include a more detailed rationale for the study. Discuss gaps in the existing literature and explain how your study addresses these gaps. | The introduction has been further expanded to provide a stronger justification for why the study is needed. | 4 |
| Operational Definitions and Consistency |  |  |
| 1. The manuscript should clearly define key terms such as "diet quality," "mental health," and "quality of transition." Consistency in how these terms are used throughout the paper is crucial for clarity. Action: Review the manuscript to ensure that all key terms are clearly defined at their first mention and used consistently throughout. | The key terms have been clearly defined by adding relevant definitions to the introduction section. | 3-4 |
| Sample Description and Representativeness |  |  |
| 1. The description of the sample could be expanded to better highlight its representativeness. How does the demographic profile of your sample compare to the broader population of first-year university students in the UK? Action: Consider including a brief discussion on the representativeness of your sample in the methods or discussion sections. | A discussion of the sample representativeness has been added to the discussion section. | 12 |
| Control Variables |  |  |
| 1. There may be other potential confounding variables that could influence the relationship between diet and mental health (e.g., socioeconomic status, pre-existing mental health conditions). Action: Discuss any additional potential confounders that were not controlled for in your analysis. If data are available, consider including these as control variables in your regression models. | The fact that there could be additional confounding variables has been discussed, with example included in the discussion section. | 13 |
| Interpretation of Statistical Significance |  |  |
| 1. While the manuscript currently focuses on statistical significance, it would be valuable to also discuss the practical significance of the findings. Action: In the discussion section, provide a more nuanced interpretation of your findings by discussing both statistical and practical significance. | The practical significance and implications of the findings have been added to the discussion section | 11 |
| Potential Impact of COVID-19 |  |  |
| 1. If the study was conducted during or after the COVID-19 pandemic, it’s important to consider how this context might have influenced the findings. Action: Include a discussion of how the timing of the study (e.g., during or post-COVID-19) might have affected the results. | A paragraph relevant to the potential effects of COVID-19 has been added to the discussion section. | 13 |
| *Reviewer 1 comments* |  |  |
| 1. The sample size is too small. not clear how it was calculated. 2. One of the limitations is that the data were self-reported, which increases the bias. 3. The assessment tools were not mentioned enough in the abstract. 4. Not all findings were discussed in the discussion 5. A pilot study for assess the reliability was not clear. 6. Tables need to be rearranged 7. The introduction and aims should be rewritten | An explanation of the sample calculation has been added.  Discussion of limitations of self reported measures has been added, as well as future recommendations.  The main assessment tools have been added to the abstract.  Discussion has been amended  Details about public involvement in survey design have been added  Changes have been made to the tables  Changes have been made to the introduction and aims as advised | 5  12-13  3  9-14  6  18-32  4 |
| *Reviewer 2 comments* |  |  |
| I believe it is a very good attempt. The authors have described all the necessary details especially in the methodology section. Results have also been presented effectively. The paper is also focused on something which is worthy of investigation. |  |  |
